# MeCP2-Related Diseases and Animal Models

**DOI:** 10.3390/diseases2010045

**Published:** 2014-01-27

**Authors:** Chinelo D. Ezeonwuka, Mojgan Rastegar

**Affiliations:** Regenerative Medicine Program, Department of Biochemistry and Medical Genetics, Faculty of Medicine, University of Manitoba, 745 Bannatyne Avenue, Winnipeg, Manitoba, R3E 0J9, Canada

**Keywords:** MeCP2, DNA methylation, Rett Syndrome, Autism Spectrum Disorders, Fetal Alcohol Spectrum Disorders, Rheumatoid arthritis, animal models

## Abstract

The role of epigenetics in human disease has become an area of increased research interest. Collaborative efforts from scientists and clinicians have led to a better understanding of the molecular mechanisms by which epigenetic regulation is involved in the pathogenesis of many human diseases. Several neurological and non-neurological disorders are associated with mutations in genes that encode for epigenetic factors. One of the most studied proteins that impacts human disease and is associated with deregulation of epigenetic processes is Methyl CpG binding protein 2 (MeCP2). MeCP2 is an epigenetic regulator that modulates gene expression by translating epigenetic DNA methylation marks into appropriate cellular responses. In order to highlight the importance of epigenetics to development and disease, we will discuss how MeCP2 emerges as a key epigenetic player in human neurodevelopmental, neurological, and non-neurological disorders. We will review our current knowledge on MeCP2-related diseases, including Rett Syndrome, Angelman Syndrome, Fetal Alcohol Spectrum Disorder, Hirschsprung disease, and Cancer. Additionally, we will briefly discuss about the existing MeCP2 animal models that have been generated for a better understanding of how MeCP2 impacts certain human diseases.

## 1. Introduction

Methyl CpG binding protein 2 (MeCP2) was first identified and characterized as a DNA binding protein that specifically binds to methyl-CpG dinucleotides [1]. The *MECP2* gene is localized on the X-chromosome and contains complex regulatory elements that control its precise expression levels [2–5]. In both human and mouse, the *MECP2*/*Mecp2* gene consists of four exons encoding for two different protein isoforms, MeCP2E1 and MeCP2E2 [6,7]. The two *Mecp2*/MeCP2 isoforms show differential temporal and brain region-specific differences in their distribution [7], and we previously showed that MeCP2E1 isoform is similarly distributed compared to total MeCP2 in murine brain cells [8]. The main functional protein domains of MeCP2 include the methyl binding domain (MBD), the transcriptional repression domain (TRD), the C-terminal domain (CTD), and the inter domain (ID) [9,10]. The MBD facilitates binding to methylated CpG dinucleotides and the preference for adjacent A/T-rich motifs [9,11]. It is also capable of binding to non-methylated DNA sequences such as the four-way DNA junctions [12,13]. The TRD domain mediates the transcriptional repressor role of MeCP2, and interacts with co-repressor complexes, such as c-Ski, mSin3A, HDAC1, and HDAC2 [5,9,14,15]. The CTD is suggested to be important for MeCP2 function and its chromatin binding activities [12]. Inter domain interactions significantly impacts MeCP2 structure and increases the stability of the protein [16,17].

MeCP2 is a nuclear protein that is mainly localized to methylated pericentromeric heterochromatin, referred to as chromocenters [18]. MeCP2 is widely expressed in several tissues, and is suggested to mediate transcriptional regulation through association with 5-methyl cytosine (5mC) and 5-hydroxymethlcytosine (5hmC), the two major types of DNA modifications [19–21]. MeCP2 binding to 5mC is associated with repressive functions through its interaction with co-repressor complexes, while binding to 5hmC is suggested to facilitate gene expression through the organization of dynamic chromatin [15,22–24]. Research studies on MeCP2 have shown diverse functions of this protein including transcriptional repression, transcriptional activation [25–27], RNA splicing [28,29], long range chromatin remodeling [30,31], modulation of chromatin architecture, and maintenance of DNA methylation [32]. This diversity in the function of MeCP2 underscores its role in many disorders.

MeCP2 is a multifunctional epigenetic regulator that is involved in transcriptional regulation as well as modulating chromatin structure [17,33]. Epigenetics control gene expression without altering the corresponding DNA sequences and impact development and disease [21,34–36]. Epigenetic mechanisms control the expression of many neurodevelopmentally important genes through chromatin remodeling, histone modifications and DNA methylation [37–41]. The prevailing view of MeCP2 as an epigenetic modulator is related to its ability to associate with different epigenetic marks (either through direct DNA binding or recruiting other transcription factors). Thus, MeCP2 acts as an ‘epigenetic reader’ that contributes in the establishment of functional states of chromatin structure, a process that is fundamental for normal cellular function. However, it is still not fully understood whether MeCP2 acts as a genome-wide epigenetic regulator or as a gene-specific transcriptional modulator, and evidence exists in favor of both mechanisms [15,42].

MeCP2 protein is highly expressed in the brain compared to other tissues [18,43,44]. The protein expression pattern within the brain follows a defined pattern, with early appearing structures, such as brainstem and thalamus, expressing the protein before more rostral structures, such as the cortex [18,43]. Temporally, MeCP2 protein expression profile is low at birth, and increases dramatically at specific time periods coinciding with the process of neuronal maturation and synaptogenesis in different parts of the brain [43,45]. Cell type-specific studies have revealed that MeCP2 levels are highest in mature neurons, while astrocytes and immature neurons express lower levels of MeCP2 [8,46,47]. The increased levels of MeCP2 expression in mature neurons are maintained throughout adulthood, implying a requirement for postmitotic neuronal function [47–49]. In neurons, MeCP2 is involved in neuronal maturation, dendrite formation, and synaptic functions [15,49,50]. Studies of mouse models lacking *Mecp2* expression in neurons further demonstrate the critical role of MeCP2 for normal brain function, especially with regard to synaptic modulation and maintenance [18,51].

Most MeCP2 mutations are *de novo* and can be grouped into three general categories; severe loss-of-function mutations, mild loss-of-function mutations and a broad group of duplications and other non-coding mutations [52,53]. Each category of mutation is associated with (a) subset(s) of neurological symptoms. Genotype-phenotype analyses have shown that there is no direct and simple correlation with the type of observed mutations and the resulting disease phenotypes. The difficulty in attributing a particular type of mutation to a specific phenotype might be in part due to the pattern of X-chromosome inactivation (XCI) [54,55].

Overall, MeCP2-related diseases that are associated with protein dysfunction are mainly characterized by cognitive impairment and intellectual disabilities [33]. However, dysregulation of the protein are also frequently observed in cases of inherited disorders and autoimmune diseases such as systemic lupus erythematosus (SLE) [56]. In this review, we will primarily focus on human diseases that are associated with MeCP2 dysfunction, and will aim to highlight the role of MeCP2 in neurological/neuropsychiatric and non-neurological disorders. In addition, animal models that have enabled a better understanding of the mechanism of MeCP2 action will be discussed.

## 2. The Role of MeCP2 in Neurological Disorders

### 2.1. Rett Syndrome

Rett Syndrome (RTT) is a progressive X-linked neurological disorder that mainly affects young girls with an incidence rate of 1:10,000–1:15,000 [15,57]. RTT is, perhaps, the most common cause of mental retardation in females [58,59]. Although it was initially thought to occur exclusively in females, males have also been identified with classic RTT [60]. In approximately 90% of RTT cases, the disease is due to *MECP2* gene mutations [61]. *MECP2* mutations that cause classical RTT in females typically result in neonatal encephalopathy and death in the first year of life in males. However, similar *MECP2* mutations may result in RTT phenotypes in males with Kleinfelter syndrome (47, XXY), or somatic mosaicism [62,63].

There is the classic RTT as well as atypical forms of RTT that deviate from the classical clinical presentation. Classic RTT is noticed primarily during infancy and can be divided into four stages that reflect the characteristic abnormalities displayed in RTT patients [64]. The first stage takes place after a period of normal development, during the first 6–18 months after birth, when developmental progression ceases and acquisition of new skills is delayed. General motor performance such as crawling, sitting, and walking are also severely impaired at this stage [58,65,66]. The second stage of RTT starts at approximately one to four years, with developmental stagnation accompanied by general growth retardation, loss of purposeful hand movements and speech, tongue protrusion, abnormal facial expression, weight loss, and gait ataxia/apraxia [58,64,67]. Autonomic dysfunction such as irregular breathing patterns, forced expulsion of air and saliva, and apnea can also be observed at this stage [68]. The duration of this stage is from weeks to approximately a year. The third RTT stage is regarded as a relatively ‘quiet’ period as stabilization of some of the symptoms occurs. However, neuromotor regression and stereotypic hand movements still persists [58,64,67]. A defining feature at this stage is the occurrence of seizures, which ranges from easily controlled to intractable epilepsy [69]. The duration of this stage is usually from years to decades [67]. The last RTT stage takes place from ages 5–15 years and beyond. Motor deterioration continues and results in loss of mobility and dependence on wheel chair. Additional abnormalities include dystonia, severe constipation, oropharyngeal dysfunction, and cardiac abnormalities [68]. As patients become older they often develop Parkinson’s-like features [64,68]. The duration of this stage varies with individuals, and usually lasts for decades.

Atypical forms of RTT deviate from classic RTT with respect to age of disease onset, clinical profile and severity of symptoms. These forms of RTT occur due to skewing of XCI, and may range from milder forms to more severe manifestations than classic RTT [70,71]. Mild variants of RTT are characterized by a later age of onset, typically occurring between one to three years of age and display mild stereotypic movements and neurologic symptoms. The preserved speech or the Zappella variant is a mild variant of RTT characterized by the ability of patients to speak a few words. Patients with this variant have a normal head size and are usually overweight [72]. The more severe variants include the congenital form (also known as the Rolando variant) that lack the early period of normal development, and a form of classical RTT with early onset seizures before the age of six months (also known as the Hanefeld variant) [73,74]. Mutations in cyclin-dependent kinase-like 5 (CDKL5) are associated with the early-onset seizure variant form of RTT, while mutations in forkhead box protein G1 (FOXG1) are associated with congenital RTT Syndrome variant [75,76].

Magnetic resonance imaging (MRI) and autopsy examination have shown that major morphological abnormalities detected in the central nervous system of RTT patients include an overall decrease in the size of the brain and of individual neurons [77]. The reduction in brain size is distributed throughout the brain and affects both white, and to a greater extent the grey matter in different brain regions [78,79]. Cortical and cerebellar degeneration have also been demonstrated to progressively occur with increasing age in RTT patients [80].

### 2.2. MECP2 Duplication Disorder

*MECP2* duplication syndrome was predicted by the observation that mice engineered to overexpress *MECP2* develop a progressive neurological disorder including stereotyped and repetitive movements, epilepsy, spasticity, hypoactivity, and early death [81,82]. This gain of function mutation occurs mostly in males [83]. Males with this disorder present clinical features, such as infantile hypotonia, severe to profound mental retardation, poor speech development, recurrent infections, epilepsy, and progressive spasticity [84,85]. The incidence rate of *MECP2* duplication disorder is estimated to be 1% of unexplained X-linked diseases and most of the reported cases are inherited [86]. However, *de novo* cases have also been documented [87]. There is considerable clinical overlap in patients with classic RTT and *MECP2* duplication disorder specifically in behavioral phenotypes, such as stereotypic hand/body movements, anxiety, and social avoidance [88]. However, in contrast to RTT, immunodeficiency is observed in patients with *MECP2* duplication disorder. It remains unclear if this phenotype occurs due to secondary effects from increased dosage of MeCP2 protein [89]. Approximately 40% of males with *MECP2* duplication reported so far have died before their 25^th^ birthday, usually from respiratory infections [90].

### 2.3. Angelman Syndrome

*MECP2* mutations that cause RTT have also been reported in cases of Angelman Syndrome (AS) [91–93]. Angelman Syndrome is primarily caused by mutations or imprinting errors of the *UBE3A* gene located on chromosome 15, and is characterized by intellectual disability; severe speech impairment and gait ataxia. Considerable phenotypic overlap exists between AS and RTT; however they differ with respect to timing of symptom onset. Angelman Syndrome has an earlier onset and patients are characterized with low hypotonicity at birth [91].

### 2.4. X-linked Mental Retardation

X-linked mental retardation (XLMR) is a genetic disorder arising from mutations or duplication of genes across the X chromosome, including the *MECP2* gene. *MECP2* point mutations have been identified in up to 2% of individuals with XLMR and duplications of the gene are also implicated in approximately 1% to 2% of XLMR cases [86,94]. *MECP2* mutations that cause RTT or severe neonatal encephalopathy are not identified in XLMR patients and *vice versa* [86,95]. In addition, the molecular lesions underlying *MECP2* duplications that result in XLMR are different from those observed in *MECP2* duplication syndrome [96]. Males with XLMR show phenotypes, such as severe intellectual disability, speech impairment, and motor abnormalities, whereas females display mild intellectual disability or are unaffected [86].

### 2.5. Severe Neonatal Encephalopathy

Males with a normal karyotype and a mutation in the *MECP2* gene present with a distinct clinical condition, and severe neonatal encephalopathy (SNE) [97]. Severe neonatal encephalopathy is a disorder characterized by a static encephalopathy, severe developmental delays and respiratory abnormalities. The mutations are usually inherited from mothers with favorable XCI skewing that display mild/no RTT symptoms. Males with SNE often die within the first years of their life due to autonomic dysfunction [95,98]. Some *MECP2* mutations that do not cause RTT in females have also been implicated in moderate to profound mental retardation, deficits in language and motor skills, obesity, autistic features, and psychiatric disorders in males [54].

### 2.6. Autism

Autism and RTT are similar developmental disorders that belong to the spectrum of autism disorders classified as pervasive developmental disorders, or Autism Spectrum Disorders (ASD) [99]. Although they are similar developmental disorders, autism differs from RTT with respect to its genetic basis. While RTT is caused by *MECP2* mutations, the genetic basis of autism is not fully clear and is proposed to involve multiple genes [100]. Mutations in the *MECP2* regulatory elements (resulting in decreased expression of the protein) are commonly associated with autism [101]. Reduced expression of MeCP2 protein has been shown to occur frequently in the frontal cortex of autistic patients and is correlated with increased *MECP2* promoter DNA methylation [102]. The silencing of autism-related genes through promoter DNA hypermethylation is commonly associated with autism, and drugs that can demethylate promoters might be useful in activating these genes [103,104]. In a recent study, we show that reduced DNA demethylation at the *Mecp2* promoter and intron 1 regulatory elements treated with Decitabine is associated with increased *Mecp2* expression. Our results provide insight on use of such drugs for future therapeutic interventions of autism [105]. Moreover, *MECP2* mutations that are associated with classic RTT have been identified in a number of autistic females who do not meet the diagnostic criteria for RTT [100]. This makes it difficult to determine if a *MECP2* mutation that is associated with autism diagnosis is a different disorder from RTT, or if both disorders are simply different representations on a spectrum associated with *MECP2* mutations. *MECP2* mutations have also been reported in patients with mild cognitive and motor difficulties and early-onset schizophrenia [106].

### 2.7. Fetal Alcohol Spectrum Disorders

Prenatal exposure to alcohol is associated with adverse effects on neurodevelopment, and results in a set of severe neurodevelopmental disorders known as fetal alcohol spectrum disorders (FASD) [107,108]. The incidence of FASD is estimated to be as high as 1 in 100 births and this disorder commonly manifests as cognitive and intellectual disabilities [108]. Accumulating evidence-implicating MeCP2 in FASD pathogenesis further reinforces the critical role of MeCP2 for central nervous system function. Several studies have demonstrated aberrant expression levels of MeCP2 in rodent FASD models, and this is suggested to be an important epigenetic determinant in FASD [109,110]. For example, prenatal exposure to ethanol has been demonstrated to significantly decrease MeCP2 expression in both prefrontal cortex and striatum of rodent offspring [109]. Research in animal models suggests that the global epigenetic changes due to ethanol are related to variations in the levels, duration as well as time of exposure [108,111]. Further supporting the potential role of MeCP2 in FASD, RTT-causing mutations have been reported in a FASD patient [112]. MeCP2 has been also shown to modulate the alcohol intake and sensitivity to alcohol, demonstrating the role of MeCP2 in alcoholism [113].

### 2.8. Huntington’s Disease

Huntington’s disease (HD) is a progressive neurodegenerative disorder that affects muscle coordination and results in cognitive decline and psychiatric disorders [114,115]. It is one of several tri-nucleotide repeat disorders caused by the length of a repeated section of a gene exceeding a normal range. Expansion of a CAG triplet repeat stretch within the *Huntingtin* gene (*HTT*) results in a mutant form of the protein, which gradually damages brain cells [114,116]. Transcriptional dysregulation has been suggested to play major roles in HD pathology, and it was recently demonstrated that the huntingtin protein (Htt) directly interacts with MeCP2 in mouse and cellular models of HD. Aberrant interactions between Htt and MeCP2 is suggested to contribute to aberrant transcription in Huntington’s disease by regulating brain-derived neurotrophic factor (BDNF) levels [117].

The implication of MeCP2 in such diverse range of neurological disorders necessitates a complete understanding of its relationship to brain development and function, as well as its interaction with other epigenetic factors that mediate dysregulation of normal epigenetic program of the brain.

## 3. Non-Neurological Disorders Associated with MeCP2

### 3.1. Cancer

Apart from to its role in neurological disorders, MeCP2 has also been shown to play a role in many cancers such as breast, colorectal, lung, liver, and prostate cancer [118–123]. MeCP2 role in cancer is related to the epigenetic regulation of cancer-related genes, particularly mechanisms that involve hypermethylation of gene promoters [118]. The growth-promoting role of MeCP2 in prostate cancer cells has been demonstrated previously, where it was shown to control mechanisms, such as cell proliferation and apoptosis [119]. In gastric cancer, the depth of invasion has been shown to be associated with MeCP2 protein levels. In gastric carcinoma cells, microRNA miR-212 was shown to suppress translation of *MECP2* transcripts, which in turn resulted in reduced depth of cellular invasion [124]. In addition, MeCP2 has been linked to other cancers, such as myeloma [125], hematological malignancies [126], ductal carcinomas [127], and cervical cancers [128].

### 3.2. Systemic Lupus Erythematosus

Systemic lupus erythematosus (SLE) is a systemic autoimmune disease that affects multiple organs. The disease predominantly affects females, with a female to male ratio that ranges from 4.3–13.6 to 1 [56,129]. Although the cause of SLE is still not clear, evidence supports an important role for abnormal T cell DNA methylation in the pathogenesis of SLE, as methylation sensitive genes show increased expression in T cells of SLE patients [130]. In active SLE T-cells, the expression of DNA methyltransferase 1 (DNMT1), the main enzyme that maintains DNA methylation during cell division, is reduced, resulting in promoter hypomethylation of these methylation-sensitive genes [56,131,132]. MeCP2 is suggested to play an important role in this process as it is critical for the epigenetic regulation of methylation-sensitive genes, and genetic polymorphisms in *MECP2* have also been identified in patients with SLE [56,133,134]. Moreover, MeCP2 associates with DNMT1, the association of which is required to maintain DNA methylation [32]. The association between MeCP2, DNMT, and methylation-sensitive genes suggests an important role for epigenetic regulation in the pathogenesis of SLE and other autoimmune diseases.

### 3.3. Rheumatoid Arthritis

Rheumatoid arthritis (RA) is a systemic autoimmune disease that results in chronic inflammation and destruction of many tissues and organs, primarily flexible synovial joints [135–137]. It is characterized by the presence of auto antibodies, which may be detected in the blood long before the onset of disease [138,139]. Increasing evidence suggests that DNA methylation and histone modifications regulate the progression of Rheumatoid arthritis. Interestingly, it has been shown that MeCP2 expression levels were up-regulated in rodent models of RA, and it is hypothesized that the increased MeCP2 protein levels play a role in the pathogenesis of RA through the canonical Wnt pathway [140,141].

### 3.4. Hirschsprung’s Disease

Hirschsprung’s disease (HSCR) is a congenital disorder of the colon characterized by the absence of certain nerve cells known as ganglion cells [142,143]. The incidence of HSCR is about 1:2000–5000, with males being affected 4 times more frequently than females [144]. The lack of ganglion cells is associated with impaired craniocaudal migration of neural crest cells, and results in severe constipation or intestinal obstruction [145,146]. Defects in the differentiation of neuroblasts into ganglion cells may also contribute to the disorder [142]. A recent study implicating MeCP2 in the pathogenesis of HSCR suggests that aberrant reduced levels of MeCP2 may play an important role in suppressing the proliferative ability of cells in patients with Hirschsprung’s disease [147]. Table 1 represents a list of MeCP2-associated diseases and the genders that are mostly affected.

## 4. Animal Models of MeCP2 Dysfunction

Several animal models have been generated in order to better understand the molecular mechanisms, progression and pathology of disorders that are associated with MeCP2 dysfunction. These include mostly rodent models (particularly mouse models) as well as zebrafish and Drosophila models. Different strategies are often employed to alter the expression and function of MeCP2 in these animal models.

### 4.1. Mecp2 Null Mouse Models

*Mecp2* knockout mice have been generated using the Cre recombinase-loxP system. These mice appear normal until approximately three to four weeks of age, when they begin to exhibit behavioral phenotypes such as unusual gait, hindlimb clasping, seizures, tremors, anxiety, learning and memory deficits, and irregular breathing. Brain neuropathology observed in *Mecp2* knockout mice is similar to that observed in RTT individuals. *Mecp2* knockout mice have smaller densely packed neurons, reduced dendritic spine density, deficits in axonal fasciculation and reduced number of mature synapses. *Mecp2* knockout mice die at approximately 10 weeks of life [150–152]. The reactivation of *Mecp2* in the brains of *Mecp2* null mice rescued many RTT-phenotypes seen in the null mice, including delayed disease progression, increased life span, deceased mortality, and restored neurological functions [153,154].

### 4.2. Mecp2 Mutant Mice

*Mecp2* mutant mice differ from the above-mentioned knockout mice as they express a truncated/mutant MeCP2 protein. One of such mutant mice is the Jaenisch strain (*Mecp2*^tm1.1jae^), generated by deletion of exon 3 of the *Mecp2* gene. Jaenisch mice display behavioral phenotypes similar to the *Mecp2* knockout mice, although the phenotype is slightly milder and the lifespan is longer (12 weeks) [82,155]. Another *Mecp2* mutant mouse model has been generated that expresses a protein truncated at amino acid 308 (*Mecp2*^308^). It is assumed that the truncated MeCP2 protein produced in these mice has residual unknown functions. *Mecp2*^308^ mice appear normal until approximately six weeks of age when they develop similar but milder neurological phenotype as *Mecp2* knockout mice [156].

### 4.3. Mecp2 Conditional-Mutant Mice

Brain-specific deletion of *Mecp2* in neural precursor cells, beginning at embryonic day 12 (*Nestin-cre* line), results in mice that display phenotypes similar to *Mecp2* knockout mice [150,155]. This suggests that MeCP2 dysfunction in the CNS is sufficient to cause phenotypes observed in RTT. Selective loss of *Mecp2* in hypothalamic and amygdala neurons (*Sim1-cre* line) results in mice that display similar abnormal physiological stress response as that observed in brain-specific conditional mice. These mice were also obese and aggressive, suggesting a role for MeCP2 in regulating social and feeding behaviors [157]. Deletion of *Mecp2* in other parts of the brain such as in brainstem neurons (*TH-cre* line) significantly affects locomotor activity, with no effect on social interaction, breathing patterns, learning and memory [158,159].

### 4.4. Mice Overexpressing MECP2

Mice overexpressing human *MECP2* at approximately twice the endogenous levels exhibit delayed neurological symptoms at approximately 10 weeks. These symptoms include enhanced motor learning and synaptic plasticity in the hippocampus. However at 20 weeks, these mice develop seizures and become hypoactive and the majority of them die by approximately one year of age [81]. Increased MeCP2 expression in other transgenic lines also results in motor abnormalities and neurological symptoms [160]. These transgenic mice mimic *MECP2* duplication syndrome that is observed in humans, and reinforces the notion of a critical requirement for precise dosage of MeCP2 protein.

### 4.5. Mouse Models Carrying Rett Syndrome-Associated Mutations

A mouse model of Rett syndrome was generated by introducing a premature STOP codon at the amino acid position 168, and resulted in a truncated MeCP2 protein (*Mecp2*^R168X^). These mice display neurological phenotypes such as hind limb clasping and breathing irregularities similar to other mouse models [161]. Another RTT mouse model is the A140V *Mecp2* mutant mice (*Mecp2*^A140V^). This MeCP2 mutation has also been described in cases of X-linked mental retardation and manic-depressive behaviors. A140V mutant mice produce a mutant MeCP2 protein that lacks the ability to bind to Alpha thalassemia/mental retardation syndrome X-linked (ATRX) protein. These mice have an apparently normal life span, and lack specific phenotypes that are displayed in other mouse models including seizures, tremors, and breathing irregularities. However they show increased cellular packing and reduced dendrite branching similar to what is observed in autopsy brains from RTT individuals [162]. MeCP2 partners with ATRX and is involved in the silencing of imprinted genes in brain [163]. Recently, a *Mecp2e1*-deficient mouse model was developed with a point mutation in exon 1 changing translational start site of the first exon from “ATG” into “TTG”. These mice show many phenotypes observed in *Mecp2* null mice including hindlimb clasping, forelimb stereotypy, and excessive grooming followed by death within 7 to 31 weeks [164]. In order to demonstrate the functions of neuronal activity-dependent phosphorylation at S80, S421 and S424, two knock-in mice models were generated; *Mecp2*^S80A^ and *Mecp2*^S421A;S424A^ abolishing the phosphorylation at S80 and S421 sites. The *Mecp2*^S80A^ mice showed reduced locomotion while opposite locomotors behaviors were seen in *Mecp2*^S421A;S424A^, providing insights on the functions of S80 phosphorylation in resting neurons and S421 in active neurons [165].

A rat model of Rett Syndrome was also generated with reduced *Mecp2* expression in the brain. This RTT rodent model revealed reduced expression of *Bdnf*, with no significant phenotypes that mimic RTT [166]. Other RTT animal models include the Drosophila and zebrafish models. The Drosophila RTT model was generated by overexpressing known RTT mutations such as R294X and R106W, which resulted in locomotary dysfunction [167]. The recently reported zebrafish model of RTT was generated by introducing a C to T transition-mutation at position 187 of the *Mecp2* coding sequence. This resulted in a nonsense mutation and a truncation of the MeCP2 protein at the position 63 (*Mecp2*Q63*). RTT zebrafish model display altered motor behaviors, although the phenotype is weaker in comparison to other *Mecp2*-deficient animal models. In contrast to MeCP2-null mouse models, *Mecp2*-null zebrafish are viable and fertile [168].

Other than animal models, *in vitro* cellular models of *Mecp2* dysfunction have also been established in order to better understand the how the effects of MeCP2 deficiency impairs normal brain function. Loss of *Mecp2* from cultured neuronal cell populations obtained from embryonic or postnatal *Mecp2*-deficient mice indicate biochemical and morphological abnormalities similar to *Mecp2* null animals [49,159,169]. Moreover, other studies have revealed putative roles of MeCP2 in astrocytes and microglia despite the low levels of MeCP2 in these cell types. *Mecp2*-deficient astrocytes and microglia have been demonstrated to produce aberrant levels of soluble factors, such as glutamate, which inhibit dendrite branching from co-cultured neurons *in vitro* [47,48,170]. In addition, loss of *Mecp2* from astrocytes has been demonstrated to affect astrocytic gap junction function, thereby resulting in their failure to adequately support dendritic development [48]. Table 2 presents an overview of animal models that are developed to study the molecular function of MeCP2.

## 5. Closing Remarks

Epigenetic regulation of gene expression is fundamental for proper organism development and function. MeCP2 is a multifunctional epigenetic regulator and the malleability in its function underscores its role in many human diseases. Since its discovery, significant progress has been made to understand its dynamic molecular properties, and increasing evidence reveals its central position in many neurological, neurodevelopmental, neuropsychiatric and non-neurological disorders. Despite tremendous progress in understanding the molecular mechanisms by which dysregulation of MeCP2 expression and function results in these disorders, we are still far from translating this knowledge towards effective therapeutic approaches. The availability of excellent animal models promises, not only hope, but also a better strategy to overcome the challenge of translational research. It is very likely that the number of diseases associated with MeCP2 dysfunction will grow rapidly in near future, thus, a better understanding of how MeCP2 functions in complex regulatory networks will pave the way for the discovery of better disease biomarkers as well as novel targets for treatments.

## Figures and Tables

**Table 1:** Human diseases associated with Methyl CpG binding protein 2 (MeCP2) and gender mostly affected.

Disease	Gender mostly affected	References
Rett Syndrome	Females (also males with kleinfelter syndrome 47 XXY, or somatic mosaicism)	[15,62,63,148]
*MECP2* duplication disorder	Males	[81–83]
Angelman Syndrome	Females	[91,93]
X-linked mental retardation	Males	[86,94,95]
Severe neonatal encephalopathy	Males	[95,97,98]
Autism	Both	[100–102]
Fetal alcohol spectrum disorders	Both (Studies from animal models only)	[109,110,113]
Huntington’s disease	Both	[117]
Early-onset schizophrenia	Both	[106]
Cancers	Both	[119–128,149]
Systemic lupus erythematosus	Females	[56,133,134]
Rheumatoid arthritis	Females	[140,141]
Hirschsprung’s disease	Males	[147]

**Table 2:** Animal models of MeCP2 dysfunction.

Animal models	Description	Phenotype	References

**Mouse**			
***Mecp2* null mouse models:**	Exon 3 and 4 deletion. MeCP2 expression and function are abolished	Unusual gait, hindlimb clasping, seizures, irregular breathing	[150,151]
*Mecp2*^tm1.1bird^			
*Mecp2*^tm1.Tam^			
*Mecp2*^lox Stop/y^ or *Mecp2*^tm2.bird^	Gene silencing by Cre recombinase insertion into intron 2. No protein is detected (behaves as a null allele)	Phenotypes similar to the null mice with abnormal behavior, RTT-like phenotypes and breathing irregularities.	[153]

***Mecp2* mutant mice:**			
*Mecp2*^tm1.1jae^	Exon 3 deletion. MeCP2 expression and function are abolished.	Neurological phenotype similar to *Mecp2* null mice, however lifespan is longer.	[155]
*Mecp2*^308^	Introduction of a premature STOP codon in exon 4. Truncated MeCP2 protein with residual unknown function	Milder neurological phenotype compared to *Mecp2* null mice	[156]

***Mecp2* conditional-mutant mice:**			
*Nestin-cre* knockout	Brain-specific deletion	Similar to Mecp2 null mice except for breathing phenotype	[150,155]
*Sim 1-cre* knockout	Selective deletion in neurons of hypothalamus and amygdala	Abnormal stress response, stranger aggression	[157]
*TH-cre* knockout	Selective deletion in dopaminergic and noradrenergic neurons.	Hypoactivity, reduced expression of tyrosine hydroxylase	[158,159]
*CamKII-cre* knockout	Forebrain-specific deletion	Impaired motor co-ordination, anxiety	[171]
*Pet1-cre* knockout	Selective deletion in serotonergic neurons	Increased aggression, hyperactivity	[159]
*Viaat-cre* knockout	Selective deletion in GABAergic neurons	Reduced lifespan, self-mutilation	[169]

**Mice overexpressing *MECP2***			
MeCP2^Tg1^	*MECP2* overexpression in all cells	Seizures, premature death, abnormal social behaviors, hypoactivity	[160]
*Tau*-*MECP2*-rescue	*MECP2* overexpression in neurons	Hypoactivity, impaired cognition	[81]

**Rett Syndrome mouse models:**			
*Mecp2*^R168X^	Premature STOP codon at amino acid 168	Hindlimb clasping, breathing irregularities	[161]
*Mecp2*^A140V^	Missense mutation that produces mutant MeCP2 protein	Normal life span, reduced dendrite branching	[162]
*Mecp2*^T308A^	Knock-in mutation that causes loss of interaction with NCoR complex	Motor abnormalities, hindlimb clasping	[172]
*Mecp2*^R306C^	Knock-in mutation that causes loss of interaction with NCoR/SMRT	Impaired motor function, hindlimb clasping	[173]
*Mecp2*^T158A^	Knock-in mutation that disrupts protein stability	Developmental regression, hypoactivity	[174]
*Mecp2-e1*	Point mutation of ATG in exon 1 to TTG	Forelimb stereotypy, hindlimb clasping, excessive grooming, and hypoactivity	[164]
*Mecp2*^S80A^	Knock-in mouse model with abolished phosphorylation at S80.	Reduced locomotion similar to that of *Mecp2* null mice and RTT patients	[165]
*Mecp2*^S421A;S424A^	Double mutant mouse model which lacks phosphorylation at both S421A and S424A	Phenotypes opposite to *Mecp2*^S80A^ mice (increased locomotion)	[165]

***Mecp2* Mouse models of phenotypic rescue**			
*Mecp2*^lox^ Stop/y;cre ^ER^	Activation of *Mecp2* gene in *Mecp2*^l^°^x St^°^p/y^ mouse model by cre-ER and Tamoxifen injections.	Rescued majority of RTT phenotypes including increased lifespan, delayed disease progression	[153]
*Mecp2*^+/ ; CAGGS LSL^ *Mecp2*	Conditional activation (rescue) of *Mecp2* gene in brain using synthetic CAGGS promoter	Partial rescue of RTT phenotypes, including delayed disease progression, reduced lethality and improved behaviors	[154]

**Rat**			
*Mecp2*-sh-1	Viral mediated RNAi-induced downregulation of *Mecp2*	Transient neurobehavioral abnormalities, reduced *Bdnf* expression in hippocampus	[166]

**Zebrafish**			
*Mecp2*Q63 *.	Nonsense mutation and a truncation of MeCP2 at position 63	Altered motor behaviors, however viable and fertile	[168]

**Drosophila**			
*GMR-Gal4:UAS-MeCP2 R106W/+.* *GMR-Gal4*:*UAS-MeCP2 R294X/+.*	Overexpression of mutant MeCP2 protein	Locomotar dysfunction, external eye disruption	[167]

## List of Abbreviations

AS: Angelman Syndrome
ASD: Autism Spectrum Disorders
ATRX: Alpha Thalassemia/Mental Retardation Syndrome X-linked
BDNF: Brain Derived Neurotrophic Factor
CDKL5: Cyclin-Dependent Kinase-Like 5
CTD: C-Terminal Domain
DNMT: DNA Methyl Transferase
FASD: Fetal Alcohol Spectrum Disorders
FOXG1: Forkhead Box Protein G1
HD: Huntington’s Disease
HSCR: Hirschsprung’s Disease
*HTT*: Huntingtin Gene
Htt: Huntingtin Protein
MBD: Methyl Binding Domain
MeCP2: Methyl CpG Binding Protein
*MECP2*: Human *MECP2* Gene
*Mecp2*: Mouse *Mecp2* Gene
miRNA: microRNA
RA: Rheumatoid Arthritis
RTT: Rett Syndrome
SLE: Systemic Lupus Erythematosus
TRD: Transcription Repression Domain
XCI: X Chromosome Inactivation
XLMR: X-linked Mental Retardation
5mC: 5-methylcytosine
5hmC: 5-hyrdoxymethylcytosine
